# Experiences of mothers of long-term surviving patients with cerebral adrenoleukodystrophy: a qualitative study

**DOI:** 10.1186/s13023-024-03424-2

**Published:** 2024-10-28

**Authors:** Yuta Koto, Nozomi Hadano, Norio Sakai

**Affiliations:** 1 Faculty of Nursing, Graduate School of Nursing, Kansai Medical University, 2-2-2 Shinmachi, Hirakata, Osaka 573-1004 Japan; 2 Department of Nursing, Faculty of Nursing, Shitennoji University, 3-2-1 Gakuenmae, Habikino, Osaka 583-8501 Japan; 3 Center for Promoting Treatment of Intractable Diseases, ISEIKAI International General Hospital, 4-14 Minamiogimachi, Kita-ku, Osaka 530-0052 Japan

**Keywords:** Adrenoleukodystrophy, Caregiver burden, Hematopoietic stem cell transplantation, Quality of life, Siblings

## Abstract

**Background:**

Adrenoleukodystrophy (ALD) is an X-linked peroxisomal disorder. Its cerebral form presents as a learning and behavioral disorder that, if untreated, leads to rapid neurological regression, disability, and death within 10 years of diagnosis. Therefore, the disease significantly impacts patients’ quality of life, making quality of life assessment crucial for effective medical treatment and care. However, no disease-specific quality of life scale exists for ALD. Therefore, we conducted qualitative research to determine the experiences of patients and their families as a preliminary step toward developing one.

**Results:**

Four mothers of patients with cerebral ALD were interviewed. Based on classification using the qualitative content analysis method, the verbatim transcripts were grouped into four themes: support needs for patients, support needs for families, the impact of treatment, and challenges within support systems.

**Conclusions:**

Support for patients and family members is required after ALD is diagnosed. In addition to addressing symptoms, daily life support and caregiving burden should be considered. Furthermore, several challenges and opportunities exist for improving treatment and support systems. Therefore, combining appropriate supporters and support systems according to the progressive and hereditary characteristics of ALD is crucial.

**Supplementary Information:**

The online version contains supplementary material available at 10.1186/s13023-024-03424-2.

## Background

### Disease overview

Adrenoleukodystrophy (ALD) is an X-linked peroxisomal disorder [1]. ALD is a progressive neurodegenerative disease manifested by the accumulation of saturated very-long-chain fatty acids due to mutations in *ABCD1* [1]. Very long-chain fatty acids accumulate in the cerebral white matter, spinal cord, and adrenal cortex [1]. ALD is a rare disorder, with an estimated incidence of 1 in 10,500 births based on data from regions with newborn screening [2]. A national survey in Japan identified 262 ALD patients, approximately 70% of whom had the cerebral form of the disease [3]. Newborn screening helps in the early detection of patients; however, the decisions regarding the management of variants of unknown significance and the treatment of female patients vary by country or region [4].

As ALD is an X-linked disease, its most prominent symptoms manifest in boys. The clinical forms of ALD can be divided into three syndromes: slowly progressive myeloneuropathy (adrenomyeloneuropathy), rapidly progressive leukodystrophy (cerebral ALD), and primary adrenal insufficiency [5]. Notably, female carriers develop myeloneuropathy in adulthood. Among the pathological types of ALD, cerebral ALD has a poor prognosis. Cerebral ALD develops at approximately 4–12 years of age as a learning and behavioral disorder, and if untreated, rapid neurological decline leads to disability for approximately 6 months and death within 5–10 years after diagnosis [6]. Consequently, male patients with advanced cerebral ALD struggle to verbally express their feelings and thoughts. Therefore, information from family members, particularly the mother, who is usually the primary caregiver, is critically important.

The prognosis for cerebral ALD is poor without treatment, and available treatment options are limited. Among these options, hematopoietic stem cell transplantation (HSCT) is the recommended treatment for ALD [1]. However, HSCT is only effective in early-diagnosed cases, in whom demyelination and neurological symptoms have not progressed, and is ineffective in advanced cases [1, 5]. In managing patients with ALD and their families, a support team, including pediatricians, genetic counselors, metabolic specialists, and neurologists, is required [5].

### Quality of life of patients with ALD

When supporting patients with ALD, a progressive disease with limited treatment options, their quality of life (QOL) should be considered. Few studies have reported on the QOL of patients with ALD [7–10]. One study of female patients used the MOS 36-Item Short-Form Health Survey (SF-36), a general QOL scale, and found that symptomatic patients had lower QOL scores compared to asymptomatic patients across all components of the SF-36 [7]. Similarly, a study using SF-36 suggested that female patients tend to have lower QOL with respect to physical functioning, vitality, and general health than those of the general population [8]. One study evaluated boys with ALD using subscales from the Neuro-QoL to assess QOL related to neurological symptoms, as well as subscales from a general QOL scale to measure physical aspects of QOL, and found that the more severe the cerebral dysfunction, the lower the QOL related to upper extremity function, physical mobility, and peer interaction [9]. In addition to neurologic symptoms and physical activity, patients with ALD also face challenges related to urinary function. One study using the SF-Qualiveen, a QOL scale for urinary disorders, also revealed no significant differences between men and women with ALD [10]. These surveys use general or partially symptom-specific QOL measures. However, the general QOL scale can assess motor function but not changes such as seizures, cognitive decline, or impaired fine motor skills. In addition, the symptoms of ALD are markedly diverse, including motor impairment, neurologic symptoms, decreased sensory function, and dysuria, making it challenging to cover a patient’s entire life with a symptom-specific scale. Furthermore, the evaluation of relationships with family members and healthcare providers is important for assessing the social QOL of patients with disabilities, a perspective that is not included in the general scale. Therefore, a disease-specific QOL scale is required to examine the QOL of patients with ALD, a progressive condition that presents with various physical symptoms, in detail.

### Scale development

Currently, no disease-specific QOL scale for ALD has been reported. Inherited metabolic leukodystrophies similar to ALD, such as Krabbe disease and metachromatic leukodystrophy, also require disease-specific QOL assessments. Langan et al. reported a scale specific to patients with leukodystrophy [11]; however, their study focused on Krabbe disease and did not necessarily address the QOL of patients with ALD. Therefore, there is a need to develop a scale specifically for ALD.

The procedure for scale development is outlined in the COnsensus-based Standards for the selection of health Measurement INstruments (COSMIN) checklist [12] and in DeVellis’ book [13]. Developing a scale involves several phases: creating a set of questions, examining the scale’s structure, and testing its validity and reliability. The development of the initial set of questions should include the findings of qualitative studies with patients or key persons and the collective opinions of experts. To date, scales have been developed based on qualitative research findings with patients and families in Fabry disease and Gaucher disease, which are rare metabolic diseases similar to ALD [14, 15]. Thus, ensuring that the findings of qualitative studies in various environments have been adequately accumulated is necessary to develop international, disease-specific measures for ALD.

### Findings of qualitative research

A qualitative systematic review of patients with leukodystrophy and their families, including those with ALD, suggested the importance of coordinating family functions, appropriately using of social support services, and providing physical care due to disease progression [16]. This systematic review included several qualitative studies on the parents of patients with ALD [17–22]. Notably, Lee et al. [18] and Feng et al. [19] are high-quality studies that reveal the experiences of families of pediatric patients with ALD in Taiwan. Evident challenges at diagnosis and in the lives of pediatric patients were observed. However, with treatment, including HSCT and supportive care, the prognosis of patients with cerebral ALD has improved, and long-term care at home beyond childhood is required.

Furthermore, the medical and social welfare environments influence the experiences of long-term surviving patients and their families. In Japan, long-term surviving patients with ALD are living in an environment where they can receive advanced medical care both in the community and at home. However, there are no existing reports on long-term survival in patients with cerebral ALD. Therefore, it is essential to gather findings from qualitative studies of long-term survivors to develop a measure that reflects the overall QOL of patients with ALD from birth through adulthood. Consequently, we aimed to conduct research to identify the experiences of patients with cerebral ALD and their families in Japan.

## Methods

### Study aim

In this study, we aimed to identify the experiences of patients with ALD and their families who are long-term survivors receiving medical care in Japan. This is part of a project to develop a new measure of QOL for patients with ALD. In developing the scale, it is necessary to aggregate literature information and collect information from patients or key persons [12, 13]. The collection and synthesis of qualitative findings related to the lives of patients with ALD will lead to the development of potential questions for future scale.

### Study design

This was a qualitative descriptive study [23], and data were collected through cross-sectional interviews with mothers familiar with the symptoms and course of ALD in patients. We employed an inductive approach for qualitative content analysis [24]. The manifest content obtained from the interviews was categorized to elicit the final scale questions.

### Participants

The participants were family members of patients with ALD. Patients with pediatric cerebral form, the most prevalent form of ALD, often find it challenging to articulate their own experiences. Thus, targeting family members, particularly the primary caregiver, is essential to gather insight into the patient’s life experiences. Family members were defined as those living with the patient. Eligibility criteria required participants to be capable of conversing in an interview setting and be at least 20 years of age. There were no restrictions on sex or role at the recruitment stage.

### Procedure for informed consent

Participants were recruited from a facility for children with disabilities and patients with ALD, as well as from family associations in Japan. We explained the study’s aim to the presidents of the facility and associations and asked them to introduce us to potential participants. Subsequently, we sent the four referred participants an explanatory document and consent form and obtained their written consent.

### Data collection: Qualitative interviews

The semi-structured online interviews were conducted in Japanese. YK conducted all the interviews. The interview included background information on the patient and family, followed by two main questions.

Q1: From the family’s perspective, what do you perceive to be the impact of ALD on the patient’s daily life? Please tell me about your experiences.

Q2: What do you perceive as the impact of ALD on your daily life as a family member? Please tell me about your experiences.

In addition to these two questions, we asked questions about living difficulties, schooling, employment, friendships, reproduction, treatment, social services, and caregiving to elicit more details from the participants’ stories (Additional file 1).

### Qualitative analysis plan

Verbatim transcripts were prepared from the interview recordings by a transcription company. Data were classified from raw responses to themes using inductive content analysis [24]. The verbatim text was separated into a series of sentences, called meaning units, each representing a single semantic content. Unnecessary parts that do not indicate semantic content, such as fillers, were removed from the meaning units. Subsequently, codes were created to indicate the content manifested in the data, and units showing similar content were grouped together. These codes were aggregated into categories and, finally, into themes based on semantic similarities.

### Role in analysis

First, YK and NH read the verbatim transcripts multiple times to fully understand their content. YK then simplified the content to create codes. These codes were consolidated into categories and themes based on content similarities. YK first classified the codes, which were then checked and revised by NH. NS also confirmed the validity of the final classification.

Native English speakers at a translation company translated the participants’ quotes in the results from Japanese to English.

### Influence of researchers

In assessing the trustworthiness of qualitative research, there is a need to describe the influence of researchers’ attributes and their relationship with participants [24, 25]. Prior to the interview, the participants were informed of the purpose and methods of the study, that their participation was voluntary, that their information would be protected, and that the content of the interview would not affect their medical nursing care. Written consent was then obtained from each participant.

Participant 1 was referred from a facility where YK had previously worked and had been directly involved in patient care. Therefore, some information may not have been verbalized because of mutual tacit understanding during the interview. During the interview, the interviewer made an effort to speak as little as possible to allow the participant’s narrative to be clear and to listen attentively in case any parts of the narrative were unclear. The second author, NH, has worked as a school nurse and is familiar with the challenges that pediatric patients face in school. The experience may have influenced a strong focus on school-related issues in the classification of codes and categories. In addition, NS was involved as a consulting physician with the patient and family associations. Therefore, although he was not a direct interviewer, he may have influenced the content of the participants’ remarks. To mitigate the influence individual preconceptions on the analysis, the codes and categories were reviewed multiple times by all three authors, aiming to ensure that personal biases did not affect the results.

## Results

### Participant demographics

Interviews were conducted with four mothers of patients with ALD (Table 1). Although the recruitment of participants included family members such as fathers and siblings, only mothers actually cooperated in the interviews. The mean age of the mothers was 50 years. One patient was a 12-year-old child, and the remaining three were adults. The patients were diagnosed with cerebral ALD between the ages of 5 and 11 years. During the interview, none of the four patients were able to stand up or adjust their positions independently. In addition, verbal communication was impossible for all patients. For supportive care, they all received tube feeding through a gastrostomy, and one had a tracheostomy. After diagnosis, they were considered to have undergone HSCT, but only two received the treatment.

**Table 1:** Characteristics of the participants at the time of interview

No	Participants	Participant’s age	Sex of patients	Patient’s age	Disease type	Age at onset	Age at diagnosis	Interview time
1	Mother	43 years	male	12 years	CALD	5 years	5 years	54 min
2	Mother	48 years	male	28 years	CALD	8 years	9 years	1 h 7 min
3	Mother	53 years	male	27 years	CALD	9 years	11 years	1 h 5 min
4	Mother	56 years	male	25 years	CALD	10 years	11 years	1 h 38 min

CALD: Cerebral adrenoleukodystrophy

Seventy-five codes were created from the verbatim transcripts of the interviews. Based on similarities in content, these codes were aggregated into 15 categories and 4 themes: support needs for patients, support needs for families, the impact of treatment, and challenges within support systems (Table 2).

**Table 2:** Classification of themes and categories

Theme	Category	Number of codes
Support needs for patients		18
	Difficulties in disease acceptance	4
	Progression of symptoms	4
	Difficulties with activities of daily living	7
	Challenges in relationships with others	3
Support needs for families		22
	Burden of caring for parents	5
	Challenges for siblings	4
	Concerns as a genetic disorder	5
	Collaboration with supporters	5
	Relationships with people outside the family	3
The impact of treatment		15
	Difficulties in diagnosis	3
	Challenges for HSCT	5
	Utilization of supportive care	3
	Expectations for future treatments	4
Challenges within support systems		20
	Utilization and dissatisfaction with support systems	11
	Barriers to schooling	9

HSCT; hematopoietic stem cell transplantation

### Support needs for patients

This theme was divided into four categories: difficulties in disease acceptance, symptom progression, difficulties with activities of daily living, and challenges in relationships with others (Table 2 and Additional file 2). For patients and their families, who had led normal lives until the onset of the disease, accepting ALD was difficult, especially as the symptoms gradually progressed. The progression of symptoms limited general life activities and affected relationships with family and friends.

### Difficulties in disease acceptance

Regarding the difficulties in accepting the disease, the psychological impact of the diagnosis on the patient and family and the gradual progression of the disease were discussed. The patient began to develop some symptoms, and after a medical examination, a diagnosis of ALD was made. The patient’s mother was shocked when the progression of ALD symptoms and prognosis were explained to her.

“It was a serious disease, and, uh, the conversation was somewhat similar to, well, someday, he might, um, die. So, um, I was feeling quite hopeless.” No. 1.

The patients became increasingly frustrated by their growing inability to perform daily activities, such as walking and changing clothes. Additionally, their mothers were troubled by the accompanying deterioration of their child’s mental health.

“There were increasingly more tasks that [my child] couldn’t do independently; and when he became mentally unstable, it became difficult to keep up with his care.” No. 2.

### Symptom progression

The mothers recognized disease progression as convulsive seizures and decreased physical functions. They became anxious because they believed that convulsive seizures triggered significant symptom progression.

“When the central venous catheter was inserted under general anesthesia, he experienced a seizure. He couldn’t even stand and, um, move his hands or legs, and just remained in his current state. Um, well within 3 d or less than a week, he was completely immobile, transitioning to his current state.” No. 1.

In addition, the symptoms continued progressing during the preparation for HSCT and ALD treatment. The mothers witnessed an increasing number of daily activities the patients could not do while accompanying them to the hospital for treatment.

“Actually, during his admission to that hospital, um, there was a gradual improvement in his condition.” No. 1.

### Difficulties with activities of daily living

The progression of symptoms led to life challenges. Initially, difficulty in walking emerged, followed by gradual impairments in oral intake and elimination. The patients were distressed by their inability to go where they wanted as they gradually lost the ability to walk.

“Ummm, I do think that it was quite challenging for him, not being able to go where he wanted. That was the point when he truly unable to move.” No. 3.

The decline in swallowing function made it difficult for patients to manage oral intake, which they had previously been able to handle. The family responded by creating a mixed diet and increasing food thickness. However, the risk of aspiration increased with progressing symptoms.

“It became increasingly difficult for him to swallow, and he would frequently cough and aspirate, which I believe must have been painful.” No. 4.

Conversely, the mothers’ narrative did not exclusively focus on negative experiences. They also reported that, despite the patients’ inability to perform previously manageable activities, there were still aspects of daily life that they looked forward to.

“Indeed, watching movies, indulging in dramas, or simply listening to music, um, brought him joy.” No. 4.

“That sort of enjoyment also happened occasionally; for example, whenever everyone is drinking together, my son would join in as well.” No. 3.

### Challenges in relationships with others

The disease affected not only the physical but also the social aspects of communication with others. The patients experienced distress due to the increasing difficulty in conversation but tried to communicate nonverbally using arm and eye movements.

“At that point, he would express his intention to call his friends. However, even on the phone, he would only produce babbling sounds, making it difficult to understand what he was saying.” No. 2.

“I noticed halfway through that he was responding with his eyes. So, um, I didn’t know what to expect at first, even during conversations. His responses also became clearer over time, and, um, even people who meet him for the first time, um, would notice that, ‘oh, he’s responding.’” No. 4.

The patients attended school and built relationships with friends until the disease onset. However, the disease made it difficult for them to continue group activities, and their peers sometimes ostracized them.

“Additionally, probably because of that, there were times when he was bullied by his friends.” No. 4.

However, mothers also spoke of positive experiences in their relationships with friends. With the support of many friends, some patients could stay in school for as long as possible and maintain their friendship.

“He’s fortunate to be surrounded by good people. So, even back during his elementary school years, everybody would help him with his bags. Yes, they would even take him on excursions, while I was there, but sort of overseeing from a distance, if that makes sense.” No. 2.

### Support needs for families

This theme comprised five categories: burden of caring for parents, challenges for siblings, concerns about a genetic disorder, challenges working with supporters, and challenges in relationships with people outside the family (Table 2 and Additional file 3). While the patients’ diagnosis and progression led to an increased care burden for family members, such as parents and siblings, the fact that ALD is a hereditary disease also affected blood relatives. In addition, the families were affected by the relationship with the supporters of the services they used to alleviate the burden of care and by the behavior limitations imposed by caregiving responsibilities.

#### Burden of caring for parents

As the disease progressed, the patients required a variety of care, primarily provided by parents. Family life became more dynamic around the patient’s care, and they needed to adjust their lifestyles.

“So, um, catering to the child’s needs is ultimately the center of everything, and uh, because of this, uh, various family events, uh, revolve around the patient. Umm, I believe that the influence centers around the patient.” No. 1.

The impact on the family was not only direct due to caregiving, but also on the family’s socialization. The mothers, who were the primary caregivers, had difficulty working. However, regarding social ties, the mothers tried to balance patient care with their work.

“Sometimes, well yes, I wouldn’t go so far as to say connections with society, but yes.” No. 3.

Moreover, the parents’ aging became challenging, as the patients grew older. The mothers were concerned about their patients’ care after their own passing and considered setting up a support system to ensure continuity of care.

“Though, well, in case we pass away first, I’m really concerned about what happens afterward. So, in any case, I make sure to establish connections in various places.” No. 4.

### Challenges for siblings

The disease affects the patients, their parents, and siblings. The mothers pondered on when to tell the siblings about the patient’s hereditary disease and the fact that the siblings might also be affected. For genetic testing of female siblings who did not develop the disease, they underwent multiple counseling sessions and made their decisions on whether to undergo testing.

“Oh yes. It was like, ‘what does she want to do?’ So, I suggested, ‘then please talk to her’ and I had them talk without my presence. It turned out, ‘she also wants to test it.’” No. 4.

#### Concerns about a genetic disorder

The patient’s hereditary disease was also a source of concern for blood relatives. The mothers feared that relatives would feel responsible for transferring the characteristics of the genetic disease.

“I haven’t been able to admit that I’m a carrier. In that case, maybe it was me or grandma, that is to say, my parents. It would imply that it was her fault, and that would really just lead to depression, the kind that would make her hit rock bottom.” No. 2.

Owing to the characteristics of X-linked diseases, the mothers may be the carriers. The mothers felt stigmatized upon learning that they might have been involved in the genetic cause of their children’s disease.

“Yes, hmmm, well he’s my child. So, it’s really, umm, disappointing. There were indeed times where I would blame myself.” No. 2.

Similarly, a mother mentioned the possibility of symptoms she experiences as a carrier. There is no information about the symptoms and prognosis of ALD carriers; however, the mother spoke about her current experience.

“Well, if I could manage my daily life, then that would be great. I believe that if I could simply get by with that, then there would be no problem at all.” No. 3.

#### Challenges working with supporters

Notably, various supporters assist patients, including doctors, nurses, and staff at daycare facilities. Mothers reported that while they could discuss their concerns and build good relationships with their supporters, there were times when they hesitated to tell them.

“Umm, maybe the diaper is a bit crooked or the person’s clothes are different. Sometimes, I think that they aren’t my child’s clothes, but I don’t say anything as it doesn’t really matter.” No. 2.

#### Challenges in relationships with people outside the family

The mothers also believe that their child’s diagnosis and progression changed their relationships with their friends and the public. For some, the relationships became estranged because they could not manage the patient’s condition.

“Um, there were people who felt so bad that they couldn’t listen to the stories and would leave, yeah. I don’t feel the need to dwell on those people. Yes, those people around me tend to get gloomy when I explain things. So, I just laugh it off.” No. 2.

### The impact of treatment

This theme had four categories: difficulties in diagnosis, challenges in HSCT, utilization of supportive care, and expectations for future treatments (Table 2 and Additional file 4). Treatment of patients begins with the difficulty of diagnosing ALD, a rare disease. Once diagnosed, the patient and family must choose whether to undergo HSCT or pursue supportive care. With limited treatment options currently available, the mothers expressed hope for the development of gene therapy and other treatment methods in the future.

### Difficulties in diagnosis

The mothers spoke about the time from the onset of symptoms to ALD diagnosis. ALD was initially mistakenly diagnosed as a developmental disability, and its symptoms progressed over time, which the mothers considered a problem of the healthcare system.

“When I showed the text from the 4th grade and document from the 6th grade, highlighting the stark differences, the doctor suggested for the first time that perhaps a magnetic resonance imaging of the brain should be considered, given my concerns as the mother. This conversation took place over a year ago.” No. 4.

### Challenges in HSCT

HSCT is recommended as a treatment option after an ALD diagnosis. However, considering the risks associated with HSCT, mothers often question whether their child should undergo transplantation. Consequently, some patients proceeded with transplantation, while others did not, due to objections from family members.

“As he continued to degenerate, both physically and intellectually, I didn’t have the courage to send him for treatment alone. After seeking a second opinion on the feasibility of living together as a family and overseeing his well-being, my family and I decided to discontinue treatment.” No. 3.

The patient’s condition progressed gradually with or without transplantation, requiring gastric lavage and tracheotomy. However, the families were hesitant and resistant to interventions that required surgery.

“At the time, we didn’t fully understand that he could no longer do tasks anymore and that he would need various tubes for support. Umm, there was a part of us that thought it would be manageable if it were just through the nose.” No. 4.

### Utilization of supportive care

The increase in supportive care also meant that more care had to be provided by the family, and the risk of accidental removal of cannulas had to be considered. A mother stated that the increased frequency of suctioning made it difficult for her to leave the house.

“It was especially so after the tracheotomy, but frequent suctioning was required. And umm, up until then, I was able to take care of him all by myself.” No. 4.

### Expectations for future treatments

The mother had high hopes for future treatments despite experiencing various difficulties in therapeutic interventions. One such hope is early detection through newborn screening. Although their children would not benefit directly, the mothers hoped that the system would be in place for children diagnosed in the future.

“Umm, there is no immediate cure for my son, but what I’m prioritizing through a patient association is the newborn screening. Yes, um, a friend’s mother also has a brother who has the disease. When I heard about their good progress after early detection and undergoing bone marrow transplantation and that he’s now playing soccer normally, that was the bottom line, just knowing that early diagnosis was better.” No. 2.

Notably, the mothers were concerned about the various complications of HSCT. Some mothers also hoped that with the introduction of gene therapy, they would no longer suffer from HSCT complications. They believed that the patients’ experiences would contribute to advancing treatment.

“When it comes to drugs, a newspaper reported about a drug developed in the United States that could prevent the wrong gene from being read, and I still remember that even now. Although further developments have been made, bone marrow transplantation remains a painful treatment.” No. 4.

#### Challenges within support systems

This theme was created by merging two categories: utilization of and dissatisfaction with support systems and challenges in working with schools (Table 2 and Additional file 5). Two aspects of the environment surrounding patients and their families were extracted: support systems and schools. Institutional challenges related to care and treatment both at home and in the hospital, as well as challenges at school—where pediatric patients spend much of their time—had a significant impact on the patient and family experience.

#### Utilization of and dissatisfaction with support systems

Children with ALD use various social services in daily life. However, the support system was complicated, and mothers were confused about the available services and how to apply for them.

“The people at the ward office would say, “call all the places that say children,” and “we’re the government, so we can’t tell you this place is good.” So, I thought, “then what should I do?” Then I was told at the hospital, “why don’t you ask the school parents association or someone else who uses it?” No. 4.

Social services also had challenges, such as limitations on the number of times they could be used and facilities that could be requested. Patients living in rural areas had limited access to available services. The mothers also complained that while there are facilities that accept older adults, only a few provide care for children with disabilities.

“There is a lot of, uh, support, um, available for older adults, but, umm, for children, adults, or disabled people, there isn’t enough support available for them.” No. 3.

### Challenges in working with schools

Schools play a critical role in supporting children with ALD. The mothers worked with the school to fulfill the patients’ needs as much as possible and were concerned about the division of responsibilities between the school and the family. Notably, while some schools specialize in educating children with special needs, the mothers felt their children should attend school with typically developing peers.

“Umm, it’s like receiving a positive, um, stimulus in various ways; when I get it, I really just enjoy it, yeah. Um, I think it’s good, and it went well.” No. 1.

“When he entered junior high school, I thought it would be impossible for him to go to a regular school.” No.4.

However, parents are sometimes required to take their children to school or chaperone them. They believed that if the school did not have special considerations, it could be good for the child but a burden on the parents.

“Additionally, um, well, medical care is still necessary. So, um, it was really difficult for a nurse to accompany him at that time. So even during school trips, I went without one, in middle school and high school.” No. 3.

The mothers wanted to work with the school and teachers to make their children comfortable. Notably, some schools were accommodating for children with disabilities by adding handrails and creating discussion forums. However, mothers were concerned that their children would be affected by any trouble with the school and adjusted how they interacted with the school accordingly.

“Like, um, when we argue, I don’t want to go to school the next day. So, um, well, I compromise on the parts that I can’t do anything about. Additionally, um, well, if I need to say something, I already feel like I’m in an inferior position. So, I express myself from that stance, and I try to avoid arguments, yeah.” No. 1.

## Discussion

### Summary of findings

This study examined the experiences of patients with ALD and their families during adolescence and adulthood. Previous reports have detailed the lives of pediatric patients with childhood cerebral ALD from the immediate onset through childhood [18]. However, with advancements in medical care, the life expectancy of children with cerebral ALD has improved, and some now reach adulthood. Our results revealed the need for improvements in patient care, support for their families, healthcare delivery, and social systems. The four themes identified in our study align with the recommendations for patient support, appropriate support systems, and coordination of family functions found in the qualitative systematic review of leukodystrophies [16]. However, a key difference between the review [16] and our research is that concerns about medical treatment do not necessarily reflect the experience of support in daily life. Feng et al. demonstrated challenges of disease acceptance, minimal information about treatment, and personal need for long-term care, similar to our study; however, their study did not address the burden of family caregiving [19]. Lee et al. showed that the characteristics of ALD, an X-linked disease, indicate the impact on family members who are carriers, which is consistent with the findings of the present study [18]. Thus, the findings of this study align with those reported in previous studies. However, we were able to identify patient and family experiences related to the Japanese medical care and environment, including matters related to the care of long-term survivors using tube feeding and ventilators, as well as school life.

In addition, because this study was conducted with Japanese mothers of patients with ALD, the Japanese healthcare system and cultural background may have influenced the results. Japan has a universal health insurance system, and treatment and support after diagnosis are covered by insurance [26]. Furthermore, for certain intractable diseases such as ALD, the system is designed to have low co-payments. The findings also suggest that public prejudice against children with disabilities has not disappeared and that mothers may be placed in a culture of shame [27]. Therefore, in terms of the transferability of the present findings, it should be noted that the results were obtained under conditions unique to Japan.

#### Support needs for patients

Understanding disease progression in ALD is vital for effective patient support. Developing appropriate care and treatment strategies for progressive symptoms is essential to maintaining the patient’s QOL [16].

An ALD diagnosis is a great shock to patients and their families. Parents whose children are diagnosed with ALD often perceive it as a sign of no hope for treatment [28]. A French retrospective study reported a median diagnosis of 7.0 years in patients with cerebral ALD [29]. Until diagnosis, the patients grow and develop like any other child and live with their families. However, the issue of patients’ acceptance of their inability to perform various activities of daily living due to the progression of their symptoms has become apparent. The cerebral form of ALD progresses rapidly if left untreated [1]. Notably, some cases of cerebral ALD have been reported in which cerebral lesion changes are arrested after onset, but the progression of symptoms resumes over time [30]. Therefore, as symptoms progress, assistance with daily activities, such as walking and elimination, and medical procedures, such as gastric lavage and tracheotomy, become necessary [3].

In addition to physical symptoms, this study suggests that patients may experience problems in their relationships with others. Interviews with patients with ALD, including the cerebral type, who could speak, also suggest that in addition to physical symptoms, the disease affects their mental and social health [31]. Parents adjust the available healthcare workers and environment to ensure that the child can live as normally as possible; however, cognitive function continues to decline [28]. Patients with metachromatic leukodystrophy, a disease similar to ALD, also face challenges with school friendships and interactions with the outside world [32]. As these references indicate, assessing the health of patients with ALD requires a multifaceted evaluation of physical, mental, and social aspects. Therefore, physicians and nurses should assist patients in maintaining friendships and supporting their daily lives. New healthcare providers must fully understand the rapid progression of the disease and its impact on mental health of patients.

### Support needs for families

The findings of this study suggest that the family members of patients with ALD are affected by the burden of caregiving and social life. Disease progression requires various types of care, including suctioning, tube feeding, and transfer, especially in adolescent patients with cerebral ALD. Therefore, family role adjustments may be necessary to address the increased care burden [16]. Parents of patients with ALD tend to be depressed and anxious in Japan and require appropriate counseling and social support to ease the burden of caregiving [33]. Therefore, physicians and nurses in outpatient and inpatient settings should assess parental burdens over time and coordinate appropriate support.

Research on the challenges faced by healthy siblings of patients with ALD is lacking. For siblings of children with severe physical and intellectual disabilities, such as cerebral ALD, there is a burden of caregiving and uncertainty about future prospects [34]. Healthy siblings are sometimes involved in the care of patients; however, mothers have considered placing patients in institutions to avoid burdening their siblings after their deaths. Therefore, a support plan should be developed for patients and their families, including both parents and siblings, recognizing that siblings who may not visit the hospital or outpatient clinic also face challenges.

Information on female carriers of ALD is insufficient; however, female carriers can present with some symptoms, and their risk increases with age [35, 36]. Furthermore, as the mothers in this study have reported, they feel guilty over their children’s ALD, believing it may be due to their own genetic contribution [18]. Parents of patients with leukodystrophy have also reported feeling excessively responsible for the testing, treatment, and healthcare of their children [37]. ALD is an X-linked disease; therefore, there is a possibility of mothers being carriers. Upon learning that ALD is hereditary, mothers often question whether their own mothers and siblings should undergo genetic testing. There is concern that genetic testing may affect the reproductive ability of siblings and parents [18]. Surveys of ALD carriers indicate that healthcare providers possess no reproductive information and understanding [38]. Therefore, when an ALD diagnosis is made, the effects on the patient, mother, and siblings must be fully considered, and doctors, nurses, and genetic counselors should provide support.

#### The impact of treatment

The first concern in the medical environment surrounding patients with ALD and their families is the difficulty and delay in diagnosis. Notably, some patients immediately underwent magnetic resonance imaging and were diagnosed; however, others were mistakenly diagnosed with developmental disorders that took more than a year from onset to diagnosis. In a national survey conducted in Japan, the mean age of diagnosis for patients with cerebral ALD was 8.3 years [3]. Due to the rarity of ALD, linking it to appropriate testing and diagnosis is difficult. The interviewees reported that, to address the lack of expertise among health care providers, parents often had to take proactive steps to clearly communicate their child’s needs, rather than extensively explaining the situation [18]. Newborn screening is useful for early diagnosis. In addition, physicians and nurses must possess knowledge of ALD and be able to refer patients to specialists when needed.

Even when a diagnosis is made, the treatment options for patients with ALD can be challenging. Only two of the patients in this study underwent HSCT, and the mothers of the other two decided not to undergo HSCT after receiving an explanation of the benefits and complications of the treatment. Regarding the long-term results of HSCT in patients with cerebral ALD, retrospective studies demonstrated improved survival rates [39–41]. However, these studies also suggested that the progression of symptoms and worsening of imaging findings before treatment might worsen after treatment. The more severe the imaging findings before HSCT, the lower the QOL and adaptive function outcomes in daily life after treatment [9, 42]. There have also been reported cases of graft-versus-host disease (GVHD) occurring after HSCT, which have led to deaths and raised concerns among the mothers in this study [39]. Therefore, HSCT should be fully explained to patients and families, their decisions should be respected, and long-term support should be provided regardless of the treatment choice. The mothers also spoke of their hopes for a new treatment alternative to HSCT. Gene therapy using lentiviral vectors for ALD has shown safety and efficacy and is expected to be utilized in the future [43]. Physiological and imaging findings. along with subjective reports from patients and families, will be crucial in developing and evaluating new treatments in the future. Therefore, developing a specific scale for ALD based on accumulating qualitative findings, such as those presented in this study, is necessary.

As symptoms progress, patients may require supportive care, such as gastrostomy or tracheostomy [3]. These supportive treatments impact both the patient’s physical stability and the family’s care burden. Similar to HSCT, a team of nurses and psychologists should be available to support the patient and family, as the decision to undergo surgery is expected to be psychologically taxing for parents.

### Challenges within support systems

As ALD progresses, patients use a variety of support in addition to hospitalization, including day services, residential facilities, and home nursing care. However, it became clear that there were challenges in assessing these services, particularly in coordinating their use. Applying for these services is complicated and burdensome for parents [37]. In addition, patients combine different types of services; however, there is no support person to serve as an integrated coordinator, and parents may have to find these services themselves [18]. This causes a heavy burden on those who are not medical or welfare professionals. A study on coordinating care for children with medical needs in Japan suggested that inadequate coordination is associated with a low QOL and a lack of timely support [44]. For patients with conditions whose degree of disability changes as the disease progresses, coordinating support at the appropriate time is essential.

Schools play an essential role in supporting children with ALD. In this study, patients initially continued attending regular schools, with healthy children, after their ALD diagnosis but later moved to special needs schools as their condition progressed. The mothers preferred their children remain in their previous schools as long as possible. However, they realized that the progression of the disease made it increasingly difficult for patients to attend regular schools, leading them to transition to special needs school. Similar to ALD, due to disease progression and rarity, patients with lysosomal disease have reported challenges such as the inability to perform certain activities at school [45]. A study of patients with ALD after HSCT showed that they were able to attend regular school, except in cases of death due to GVHD or progression [46]. Therefore, patients with ALD need to be able to attend school and maintain friendships and social lives before the onset of the disease. Achieving this requires understanding and support from the surrounding community, including teachers, as well as addressing the symptoms and treatment.

Furthermore, as reported by the mothers, the support system for patients with ALD attending school was inadequate, requiring parents to be present at school to care for their children. A survey of patients with ALD in Japan highlighted the need for assistance in attending school as a concern [47]. As symptoms progress and multiple disabilities develop, patients may require after-school daycare or home healthcare. The exchange of information and the maintenance of consistency of care between the supporters of these facilities, schoolteachers, and parents are essential in supporting the patients [48].

### Future scale development

The findings of this study were obtained from the mothers and may reflect the experiences of the proxy. Ideally, the development of patient-reported outcomes should be based on findings derived from the patient’s own experiences [12]. However, the progression of ALD often makes it difficult for patients to express their own experiences [6]. Therefore, we believe that proxy narratives can be used to extract symptoms and life challenges related to disease progression. Conversely, the findings in this study do not indicate that only proxy-reported outcomes will be developed. For patients unable to speak due to disease progression, the outcome would be reported by a proxy; however, for patients who are in the early stages of progression or whose condition has been partially controlled by early diagnosis, self-reporting would still be possible. A scoping review of patient-reported outcomes in lysosomal disease, a metabolic disease similar to ALD, indicates that proxy- and patient-reported measures have been developed and studied [49]. Future measure development and operation will need to look at both patient- and proxy-reported forms.

### Limitations

This study has some limitations. First, the number of participants in this study was small. In the trustworthiness of content analysis methods, confirming data saturation is a matter of credibility [24]. Normally, we should confirm that the data obtained by increasing the number of participants would not lead to the creation of new categories or themes; however, we were unable to do this due to the rarity of ALD. Nevertheless, whenever possible, information related to the description of the research process and the investigator’s preconceived notions were clearly stated in the paper to increase credibility. However, because ALD is rare and the cerebral form even rarer, the findings of this study will be useful even if data saturation is not reached.

Second, only mothers participated in the interviews. In Japan, mothers are the primary caregivers, and we believe we were able to gather information on the overall life of the patients. However, it may be necessary to clarify the experiences of fathers and siblings to understand the X-linked inherited disease and the impact of caregiving on the lives of the entire family.

Further, the present findings are influenced by the Japanese healthcare system and cultural background. It is suggested that Japanese culture has influenced the public’s view of handicapped children and their mothers’ values [27]. The development of internationally usable scales will require the integration of knowledge about patient experiences beyond the influence of regional characteristics.

Finally, the authors involved in the data collection and analysis had experience treating and caring for patients with ALD. While this has a positive impact on the depth of interview content in terms of rich data collection, the interview content may have been influenced by the values of the healthcare providers. Therefore, in the next step—the development of questions for the scale—patients or family members will need to participate, ensure that opinions are not biased toward the medical perspective [50].

## Conclusions

We collected qualitative data regarding the lives of Japanese patients with cerebral ALD from the mothers’ perspective. Support from patients and family members is required after ALD diagnosis. Therefore, in addition to addressing symptoms, daily life support and the burden of caregiving should be considered. Furthermore, several challenges and opportunities exist for improving treatment and support systems. Identifying the experiences of patients with ALD and their mothers was a valuable source of information for understanding and addressing both their support needs and symptom management. Combining appropriate supporters and support systems based on the progressive and hereditary characteristics of ALD is crucial. Furthermore, the codes and categories obtained in this study could be used to develop candidate questions for the development of an international disease-specific QOL scale. In particular, issues related to supportive care, coordination of community care, and schools, which have not been identified in previous studies, would greatly assist in the development of items for the scale.

## Electronic Supplementary Material

Below is the link to the electronic supplementary material.

Supplementary Material 1

## Data Availability

This study data cannot be shared openly to protect study participant privacy. The data generated and/or analyzed in the current study are available from the corresponding author upon reasonable request and with the approval of the ethics committee.

## Abbreviations

ALD: Adrenoleukodystrophy
HSCT: Hematopoietic stem cell transplantation
QOL: Quality of life
GVHD: Graft-versus-host disease

## References

[1] Kemp S, Huffnagel IC, Linthorst GE, Wanders RJ, Engelen M. <ArticleTitle Language=“En”>Adrenoleukodystrophy - neuroendocrine pathogenesis and redefinition of natural history. Nat Rev Endocrinol. 2016;12:606–15. 27312864 10.1038/nrendo.2016.90

[2] Priestley JRC, Adang LA, Drewes Williams S, Lichter-Konecki U, Menello C, Engelhardt NM, et al. Newborn screening for X-linked adrenoleukodystrophy: review of data and outcomes in Pennsylvania. Int J Neonatal Screen. 2022;8:23. 35466195 10.3390/ijns8020024 PMC9036281

[3] Koto Y, Sakai N, Lee Y, Kakee N, Matsuda J, Tsuboi K, et al. Prevalence of patients with lysosomal storage disorders and peroxisomal disorders: A nationwide survey in Japan. Mol Genet Metab. 2021;133:277–88. 34090759 10.1016/j.ymgme.2021.05.004

[4] Videbæk C, Melgaard L, Lund AM, Grønborg SW. Newborn screening for adrenoleukodystrophy: International experiences and challenges. Mol Genet Metab. 2023;140:107734. 37979237 10.1016/j.ymgme.2023.107734

[5] Engelen M, van Ballegoij WJC, Mallack EJ, Van Haren KP, Kohler W, Salsano E, et al. International recommendations for the diagnosis and management of patients with adrenoleukodystrophy: A consensus-based approach. Neurology. 2022;99:940–51. 36175155 10.1212/WNL.0000000000201374 PMC9687408

[6] Zhu J, Eichler F, Biffi A, Duncan CN, Williams DA, Majzoub JA. The changing face of adrenoleukodystrophy. Endocr Rev. 2020;41:577–93. 32364223 10.1210/endrev/bnaa013 PMC7286618

[7] Schäfer L, Roicke H, Bergner CC, Köhler W. Self-reported quality of life in symptomatic and asymptomatic women with X-linked adrenoleukodystrophy. Brain Behav. 2023;13:e2878. 36748403 10.1002/brb3.2878 PMC10013936

[8] Engelen M, Barbier M, Dijkstra IM, Schur R, de Bie RM, Verhamme C, et al. X-linked adrenoleukodystrophy in women: a cross-sectional cohort study. Brain. 2014;137:693–706. 24480483 10.1093/brain/awt361

[9] Beckmann NB, Miller WP, Dietrich MS, Orchard PJ. Quality of life among boys with adrenoleukodystrophy following hematopoietic stem cell transplant. Child Neuropsychol. 2018;24:986–98. 28934891 10.1080/09297049.2017.1380176

[10] Hofereiter J, Smith MD, Seth J, Tudor KI, Fox Z, Emmanuel A, et al. Bladder and bowel dysfunction is common in both men and women with mutation of the ABCD1 gene for X-linked adrenoleukodystrophy. JIMD Rep. 2015;22:77–83. 25763509 10.1007/8904_2015_414 PMC4486267

[11] Langan TJ, Barczykowski A, Jalal K, Sherwood L, Allewelt H, Kurtzberg J, et al. Survey of quality of life, phenotypic expression, and response to treatment in Krabbe leukodystrophy. JIMD Rep. 2019;47:47–54. 31240167 10.1002/jmd2.12033 PMC6498827

[12] Terwee CB, Prinsen CAC, Chiarotto A, Westerman MJ, Patrick DL, Alonso J, et al. COSMIN methodology for evaluating the content validity of patient-reported outcome measures: a Delphi study. Qual Life Res. 2018;27:1159–70. 29550964 10.1007/s11136-018-1829-0 PMC5891557

[13] DeVellis RF, Thorpe CT. Scale development: Theory and applications. 5th ed. CA: SAGE publications. Chapter 5, Guidelines in scale development. pp. 91–135.

[14] Koto Y, Yamashita W, Lee Y, Hadano N, Kokubu C, Sakai N. Development and validation of a disease-specific quality of life scale for adult patients with Fabry disease in Japan. J Patient Rep Outcomes. 2022;6:115. 36394676 10.1186/s41687-022-00525-z PMC9672224

[15] Narita A, Koto Y, Noto S, Okada M, Ono M, Baba T, et al. Development and evaluation of a patient-reported outcome measure specific for Gaucher disease with or without neurological symptoms in Japan. Orphanet J Rare Dis. 2024;19:11. 38183145 10.1186/s13023-023-02996-9 PMC10770997

[16] Koto Y, Ueki S, Yamakawa M, Sakai N. Experiences of patients with metachromatic leukodystrophy, adrenoleukodystrophy, or Krabbe disease and their family members: a qualitative systematic review. JBI Evid Synth. 2024;22:1262–302. 38533650 10.11124/JBIES-23-00303 PMC11230659

[17] Schwan K, Youngblom J, Weisiger K, Kianmahd J, Waggoner R, Fanos J. Family perspectives on newborn screening for X-linked adrenoleukodystrophy in California. Int J Neonatal Screen. 2019;5:42. 33073000 10.3390/ijns5040042 PMC7510238

[18] Lee TY, Li CC, Liaw JJ. The lived experience of Taiwanese mothers of a child diagnosed with adrenoleukodystrophy. J Health Psychol. 2014;19:195–206. 23300045 10.1177/1359105312467388

[19] Feng JC, Wu WW, Chwo MJ, Liang SY, Cheng SF. [The long-term care experiences and care needs of parents caring for children with adrenoleukodystrophy]. Hu Li Za Zhi. 2019;66:27–37. Chinese. 30648243 10.6224/JN.201902_66(1).05

[20] Forrest LE, Curnow L, Delatycki MB, Skene L, Aitken MA. Health first, genetics second: Exploring families’ experiences of communicating genetic information. Eur J Hum Genet. 2008;16:1329–35. 18493266 10.1038/ejhg.2008.104

[21] Marly Akemi Shiroma N, Roseney B, Laura Filomena Santos de A, Leandro Felipe M. Ways of weaving networks for the care by the family that is experiencing the chronic condition by adrenoleukodystrophy. Ciência Cuidado e Saúde. 2012;11:156 – 65.

[22] Santos RNC, Bellato R, de Araújo LFS, de Almeida KBB, de Souza ÍP. Men’s position in family care on situations of chronic illness. Revista da Escola de Enfermagem. 2018;52:e03398. 10.1590/S1980-220X2017046703398 30540127

[23] Sandelowski M. What’s in a name? Qualitative description revisited. Res Nurs Health. 2010;33:77–84. 20014004 10.1002/nur.20362

[24] Kyngäs H, Kääriäinen M, Elo S. The trustworthiness of content analysis. In: Kyngäs H, Mikkonen K, Kääriäinen M, editors. The Application of content analysis in nursing science research. Springer; 2020. pp. 41–8.

[25] Lockwood C, Porritt K, Munn Z, Rittenmeyer L, Salmond S, Bjerrum M et al. Chapter 3: Systematic reviews of qualitative evidence In: Aromataris E, Lockwood C, Porritt K, Pilla B, Jordan Z, editors. JBI Manual for Evidence Synthesis. JBI; 2024. https://synthesismanual.jbi.global. Accessed 9 July 2024.

[26] Sakamoto H, Rahman M, Nomura S, Okamoto E, Koike S, Yasunaga H et al. Japan health system review. Health Syst Transit. New Delhi: World Health Organization, Regional Office for South East Asia. 2018;8:23–42. https://apps.who.int/iris/handle/10665/259941

[27] Sato T. Creation of care through communication by nurses, welfare workers, and persons (children) with profound intellectual multiple disabilities at a day care center: emancipation from the Japanese shame culture. Adv Nurs Sci. 2022;45:E69–93. 10.1097/ANS.0000000000000386 PMC9048620 34121065

[28] Piercy H, Nutting C. The experiences of parents of children diagnosed with cerebral adrenoleukodystrophy. Child Care Health Dev. 2024;50:e13184. 37850425 10.1111/cch.13184

[29] Sevin C, Hatteb S, Clément A, Bignami F, Chillotti L, Bugnard F, et al. Childhood cerebral adrenoleukodystrophy (CCALD) in France: epidemiology, natural history, and burden of disease - A population-based study. Orphanet J Rare Dis. 2023;18:238. 37563635 10.1186/s13023-023-02843-x PMC10416383

[30] Mallack EJ, van de Stadt S, Caruso PA, Musolino PL, Sadjadi R, Engelen M, et al. Clinical and radiographic course of arrested cerebral adrenoleukodystrophy. Neurology. 2020;94:e2499–507. 32482842 10.1212/WNL.0000000000009626 PMC7455338

[31] Varma A, Weinstein J, Seabury J, Rosero S, Dilek N, Heatwole J, et al. Patient-reported impact of symptoms in adrenoleukodystrophy (PRISM-ALD). Orphanet J Rare Dis. 2024;19:127. 38504253 10.1186/s13023-024-03129-6 PMC10953228

[32] Koto Y, Yamashita W, Sakai N. Impact on physical, social, and family functioning of patients with metachromatic leukodystrophy and their family members in Japan: A qualitative study. Mol Genet Metab Rep. 2024;38:101059. 38469094 10.1016/j.ymgmr.2024.101059 PMC10926226

[33] Kuratsubo I, Suzuki Y, Shimozawa N, Kondo N. Parents of childhood X-linked adrenoleukodystrophy: high risk for depression and neurosis. Brain Dev. 2008;30:477–82. 18430533 10.1016/j.braindev.2007.12.012

[34] Kruithof K, Ijzerman L, Nieuwenhuijse A, Huisman S, Schippers A, Willems D, et al. Siblings’ and parents’ perspectives on the future care for their family member with profound intellectual and multiple disabilities: A qualitative study. J Intellect Dev Disabil. 2021;46:351–61. 10.3109/13668250.2021.1892261 39818599

[35] Schirinzi T, Vasco G, Aiello C, Rizzo C, Sancesario A, Romano A, et al. Natural history of a cohort of ABCD1 variant female carriers. Eur J Neurol. 2019;26:326–32. 30295399 10.1111/ene.13816

[36] Huffnagel IC, Dijkgraaf MGW, Janssens GE, van Weeghel M, van Geel BM, Poll-The BT, et al. Disease progression in women with X-linked adrenoleukodystrophy is slow. Orphanet J Rare Dis. 2019;14:30. 30732635 10.1186/s13023-019-1008-6 PMC6367840

[37] Yazdani PA, St-Jean ML, Matovic S, Spahr A, Tran LT, Boucher RM, et al. The experience of parents of children with genetically determined leukoencephalopathies with the health care system: A qualitative study. J Child Neurol. 2023;38:329–35. 37225698 10.1177/08830738231176672 PMC10338692

[38] Choi J, Kane T, Propst L, Spencer S, Kostialik J, Arjunan A. Not just carriers: experiences of X-linked female heterozygotes. J Assist Reprod Genet. 2021;38:2757–67. 34333720 10.1007/s10815-021-02270-6 PMC8581108

[39] Kühl JS, Kupper J, Baqué H, Ebell W, Gärtner J, Korenke C, et al. Potential risks to stable long-term outcome of allogeneic hematopoietic stem cell transplantation for children with cerebral X-linked adrenoleukodystrophy. JAMA Netw Open. 2018;1:e180769. 30646031 10.1001/jamanetworkopen.2018.0769 PMC6324299

[40] Kühl JS, Suarez F, Gillett GT, Hemmati PG, Snowden JA, Stadler M, et al. Long-term outcomes of allogeneic haematopoietic stem cell transplantation for adult cerebral X-linked adrenoleukodystrophy. Brain. 2017;140:953–66. 28375456 10.1093/brain/awx016

[41] Raymond GV, Aubourg P, Paker A, Escolar M, Fischer A, Blanche S, et al. Survival and functional outcomes in boys with cerebral adrenoleukodystrophy with and without hematopoietic stem cell transplantation. Biol Blood Marrow Transpl. 2019;25:538–48. 10.1016/j.bbmt.2018.09.036 30292747

[42] Pierpont EI, McCoy E, King KE, Ziegler RS, Shanley R, Nascene D, et al. Post-transplant adaptive function in childhood cerebral adrenoleukodystrophy. Ann Clin Transl Neurol. 2018;5:252–61. 29560371 10.1002/acn3.526 PMC5846389

[43] Eichler F, Duncan C, Musolino PL, Orchard PJ, De Oliveira S, Thrasher AJ, et al. Hematopoietic stem-cell gene therapy for cerebral adrenoleukodystrophy. N Engl J Med. 2017;377:1630–8. 28976817 10.1056/NEJMoa1700554 PMC5708849

[44] Matsuzawa A, Shiroki Y, Arai J, Hirasawa A. Care coordination for children with medical complexity in Japan: Caregivers’ perspectives. Child Care Health Dev. 2020;46:436–44. 32246855 10.1111/cch.12767

[45] de Dios García-Díaz J, López-Rodríguez M, Morales-Conejo M, Riera-Mestre A. Minority Diseases Working Group from the Spanish Society of Internal M. Understanding the ecosystem of patients with lysosomal storage diseases in Spain: a qualitative research with patients and health care professionals. Orphanet J Rare Dis. 2022;17:17. 35031060 10.1186/s13023-021-02168-7 PMC8760689

[46] Gassas A, Raiman J, White L, Schechter T, Clarke J, Doyle J. Long-term adaptive functioning outcomes of children with inherited metabolic and genetic diseases treated with hematopoietic stem cell transplantation in a single large pediatric center: Parents’ perspective. J Pediatr Hematol Oncol. 2011;33:216–20. 21336168 10.1097/MPH.0b013e3182050945

[47] Sakurai K, Ohashi T, Shimozawa N, Joo-Hyun S, Okuyama T, Ida H. Characteristics of Japanese patients with X-linked adrenoleukodystrophy and concerns of their families from the 1st registry system. Brain Dev. 2019;41:50–6. 30077509 10.1016/j.braindev.2018.07.007

[48] Koto Y, Tomozawa M, Sato T, Niinomi K, Sakai N, Nagai T. Supporters’ experiences of sensory characteristics of children with profound intellectual and multiple disabilities in after-school daycare centres: A qualitative study. Nurs Open. 2023;10:7826–38. 37823349 10.1002/nop2.2031 PMC10643818

[49] McDool E, Powell P, Carlton J. Measuring health related quality of life (HRQoL) in Lysosomal Storage Disorders (LSDs): a rapid scoping review of available tools and domains. Orphanet J Rare Dis. 19;2024:252. 10.1186/s13023-024-03256-0 PMC11225496 38965628

[50] Wiering B, de Boer D, Delnoiji D. Patient involvement in the development of patient-reported outcome measures: a scoping review. Health Expect. 2017;20:11–23. 26889874 10.1111/hex.12442 PMC5217930

