# Supplementary material for: Experiences of mothers of long-term surviving patients with cerebral adrenoleukodystrophy: a qualitative study
Source: Orphanet J Rare Dis. 2024 Oct 28;19:401. doi: 10.1186/s13023-024-03424-2 (PMC11520452; doi:10.1186/s13023-024-03424-2)
Supplement: Supplementary file 1 — Supplementary Material 1 [file 13023_2024_3424_MOESM1_ESM.docx]

**Additional file 1: Interview guide**

Background of the participants and patients

Age, sex, and relationships with patients

What was the patient’s age at the time of diagnosis?

What type of disease was the patient diagnosed with?

What was the course of the patient's symptoms?

What treatment has the patient received so far?

Main question

From the family's perspective, what do you perceive as the impact of ALD on the patient's daily life? Please tell me about your experiences.

What do you perceive as the impact of ALD on your daily life as a family member? Please tell me about your experiences.

Subsidiary question+

Have there been times when you think your symptoms have made life more difficult?

What impact does this have on the patient’s life?

What impact does this have on your employment?

Do you experience difficulties in your or your patient’s relationships with friends or colleagues?

What impact does this have on childbirth?

How does the treatment method affect your life or that of your patient?

Are there any medical or social services currently used by your patient?

As a family member of the patient, did you feel restricted in your daily life or burdened by caregiving?

Do you have any expectations or concerns about the new treatments?

What concerns do you have regarding your future?

+Subsidiary questions were asked as appropriate if the participant seemed reluctant to answer the main question or if the interviewee thought that only brief answers had been given and sufficient data had not been obtained.

**Additional file 2: Categories and codes of the theme “Support needs for patients.”**

| Category | code |
| --- | --- |
| Difficulties in disease acceptance | |
|  | Shock of diagnosis |
|  | Explanation to patients |
|  | Conflicts about not being able to |
|  | Seek alternative therapies |
| Progression of symptoms | |
|  | Concerns about worsening conditions |
|  | Progress while waiting for treatment |
|  | Appearance of seizures |
|  | Stability of condition |
| Difficulties with activities of daily living | |
|  | Difficulty walking |
|  | Difficulty with oral intake |
|  | Visual challenges |
|  | Incontinence problems |
|  | Bathing issues |
|  | Difficulty in holding trunk |
|  | Daily delights |
| Challenges in relationships with others | |
|  | Difficulty in conversation |
|  | Relationships with friends |
|  | Use of Hearing |

**Additional file 3: Categories and codes of the theme “Support needs for families.”**

| Category | code |
| --- | --- |
| Burden of caring for parents | |
|  | Caring by parents |
|  | Cooperation of fathers |
|  | Difficulty in going to the hospital |
|  | Challenges of parental employment |
|  | Aging parental issues |
| Challenges for siblings | |
|  | Hereditary concerns for siblings |
|  | Explanation to siblings |
|  | Conflicts in siblings |
|  | Support for siblings |
| Concerns as a genetic disorder | |
|  | Mother's concerns as a carrier |
|  | Worries about heredity to the next child |
|  | Early detection of blood relatives |
|  | Difficulty in communicating with relatives |
|  | Relation problems with relatives |
| Collaboration with supporters | |
|  | Relationships with physicians |
|  | Relationships with nurses |
|  | Consultation with a specialist |
|  | Communicate care instructions to facilities |
|  | Satisfaction with medical care and support |
| Relationships with people outside the family | |
|  | Connection to the world |
|  | Relationships with mom friends |
|  | Participation in patient groups |

**Additional file 4: Categories and codes of the theme “The impact of treatment.”**

| Category | code |
| --- | --- |
| Difficulties in diagnosis | |
|  | Diagnosis delay |
|  | Suspected developmental disorder |
|  | Receiving a complete medical examination |
| Challenges for HSCT | |
|  | Transplant decision |
|  | Implementation of transplantation |
|  | Post-transplant complications |
|  | Concern about infectious diseases |
|  | Progression after transplantation |
| Utilization of supportive care | |
|  | Prevention of pneumonia |
|  | Trouble with tracheostomy |
|  | Creation and use of gastric lavage |
| Expectations for future treatments | |
|  | Expectations for future treatment and systems |
|  | Expectations for gene therapy |
|  | Expectations for early diagnosis |
|  | Hope for the future gained from patients |

HSCT: hematopoietic stem cell transplantation

**Additional file 5: Categories and codes of the theme “Challenge within support systems.”**

| Category | code |
| --- | --- |
| Utilization and dissatisfaction with support systems | |
|  | Requests to administration |
|  | Slowness of administrative procedures |
|  | Lack of places for consultation |
|  | Limitations of service utilization |
|  | Fewer facilities available |
|  | Securing the number of facilities involved |
|  | Use of daycare facilities |
|  | Transportation to and from facilities |
|  | Use of short-term residential facilities |
|  | Admission to a facility |
|  | Challenges in using helpers |
| Barriers to schooling | |
|  | Academic difficulties |
|  | Cooperation with school |
|  | Relationship with school teachers |
|  | Dissatisfaction with responses that are not developmentally appropriate |
|  | Expectations of going to school in the school district |
|  | Challenges in getting to and from school |
|  | Parental chaperones to school |
|  | Transfer to a school for special needs |
|  | Acceptance of visiting education |
